# Endoscopic Gastric Sleeve: A Review of Literature

**DOI:** 10.7759/cureus.36353

**Published:** 2023-03-19

**Authors:** Basil N Nduma, Kelly A Mofor, Jason T Tatang, Chukwuyem Ekhator, Solomon Ambe, Ekokobe Fonkem

**Affiliations:** 1 Internal Medicine, Merit Health Wesley, Hattiesburg, USA; 2 Gastroenterology, Paul L Foster School of Medicine, El Paso, USA; 3 Gastroenterology, Sam Houston State University, Huntsville, USA; 4 Neuro-Oncology, New York Institute of Technology, College of Osteopathic Medicine, Old Westbury, USA; 5 Neurology, Baylor Scott & White Health, Mckinney, USA; 6 Neuro-Oncology, Baylor Scott & White Health, Temple, USA

**Keywords:** bariatric surgery, endoscopic sleeve gastrectomy, obesity, endoscopic, gastric sleeve

## Abstract

For morbid obesity, one of the treatment options that have been deemed the most effective is bariatric surgery. Specifically, endoscopic sleeve gastrectomy (ESG) has emerged as one of the minimally invasive procedures for weight loss to be developed recently. In this procedure, there is the endoscopic placement of sutures in a quest to ensure reductions in the stomach volume. In this review, the main aim was to review the literature concerning ESG’s efficacy and safety. Secondary sources of data were used and electronic databases were searched to identify articles focused on assessing the safety or efficacy of ESG. They included several databases such as Clinicaltrials.gov, Embase (Excerpta Medica Database), and MEDLINE (Medical Literature Analysis and Retrieval System Online, or MEDLARS Online) to select relevant articles. Both the titles and abstracts of the articles were used to determine their inclusion or exclusion from the current review. Additionally, some keywords were used to search and obtain relevant articles such as: ESG, obesity, bariatric surgery, and total body weight loss. This review relied on the Preferred Reporting Items for Systematic Reviews and Meta-Analyses (PRISMA) framework for the identification of articles, screening, determination of eligibility, and inclusion and exclusion as deemed appropriate. From the findings, the review established that ESG is effective when used as an alternative intervention for obesity. The beneficial effects are felt particularly in terms of the procedure’s capacity to ensure that the total body weight loss mean percentage is significant. Apart from the benefit of ensuring weight loss, ESG was also found to impair gastric emptying, pose metabolic effects that are key to controlling obesity-associated metabolic dysregulation, and the ability to increase satiety. However, the procedure was documented to yield a few adverse events in some studies. Some of the notable adverse events include pulmonary embolism, potential pneumoperitoneum, and possible post-procedure leak in the posterior aspect of the gastric fundus as sutures exert tension and also cause thin walls. Emerging as a minimally-invasive procedure, ESG is a cost-effective alternative through which weight loss can be achieved significantly in obese populations. It leads to a slowdown of gastric emptying, causes an increase in satiety, and leads to an improvement in the metabolic profile. Therefore, for obese individuals not undergoing bariatric surgery, ESG can be an ideal treatment option, including individuals in need of a bridge to surgery and also those diagnosed with moderate obesity. Overall, when it comes to the management of obesity, this review established that ESG provides a paradigm shift targeting existing therapeutic gaps.

## Introduction and background

Around the world, one of the healthcare system burdens involves obesity as an epidemic with associated co-morbidities. With global escalations in the rates of obesity, bariatric surgery has been documented to exhibit limitations in attending to the needs of all suitable individuals [1]. Also, most individuals diagnosed with the condition have suffered from the disease’s burden with no prospects of getting actual treatment. Hence, more and more scholarly investigations have strived to uncover alternative procedures through which the perceived limitations associated with bariatric surgery could be countered. The key emphasis has also been on less invasive procedures to ensure the needs of patients with mild obesity and overweight issues are addressed, especially those who fail to satisfy the operative treatment’s eligibility criteria [2].

Indeed, one such alternative that has been investigated to determine its efficacy for use in the place of bariatric surgery is endoscopic sleeve gastroplasty (ESG). Also referred to as the Apollo technique, ESG operates by creating an apposition of the posterior, greater curvature, and anterior gastric body wall via the use of a full-thickness endoscopic suturing device [3]. Hence, through ESG, an endoscopist engages in the alteration of the stomach’s shape into a tubular version from the initial bean-like fashion [4].

Globally, the high prevalence of obesity has been concerning, especially because of the condition’s associated metabolic effects such as the risk of mortality, poor overall quality of life, psychosocial and functional disability, obstructive sleep apnea, cardiovascular disease, and type 2 diabetes mellitus [5]. Previously, bariatric surgery alongside lifestyle interventions has proved effective in obesity treatment especially due to the capacity to achieve and maintain a substantial loss of weight, besides comorbidity improvement [6]. Around the world, about 96% of bariatric surgical procedures are in the laparoscopic form. However, forms of bariatric surgical procedures such as laparoscopic sleeve gastrectomy (LSG) tend to cause adverse events in as high as 10-17% of the cases, with about 0.3% of the procedures associated with postoperative mortality [7]. LSG has been superior to other approaches for weight loss but its failure rate stands between 15% and 50%, besides being associated with weight regain of 5% and 70% at two years and six years, respectively [8]. In response, there has been a growing demand for alternative procedures that could lead to effective loss of weight while yielding fewer serious adverse effects, that may come with more accessible eligibility criteria.

This review sought to examine some of the experimental investigations that employed the ESG procedure, their designs, results, recommendations, and the implication for clinical environments. The motivation was to seek to shed light on the efficacy of the ESG procedure, any adverse events with which it may be associated, and the future outlook of the healthcare industry in the wake of adopting this weight loss approach, as well as its safety, the impact on the quality of life of patients, and weight loss effectiveness.

## Review

Materials and methods

This is a review of the literature and the initial methodological consideration involved the eligibility criteria. The review included articles that were not only original but also peer-reviewed, focusing on ESG implementation in obesity treatment. The articles were full-text, English publications, with publication dates between 2012 and 2022. In cases where the same subjects were included in multiple studies, only the studies with the largest number of subjects were utilized. Additionally, studies that featured these same subjects but with distinct control groups were also considered. The review included case reports, case series, case-control studies, cohort studies, prospective non-randomized trials, and randomized clinical trials, and excluded protocol studies, brief reports, letters, comments on papers, and guidelines. In relation to the participants, this review selected articles that focused on persons of all ages, as long as ESG-based treatment had been involved. Animal studies were excluded, as well as studies involving patients who had undergone prior abdominal surgical procedures. Considering the duration of studies, both short-term and long-term outcomes of ESG were of interest. As such, articles that were included were those that reported outcomes related to weight loss and further follow-up of at least two months. With adverse-event data generally limited, the aforementioned follow-up periods were deemed acceptable.

On the factor of intervention as part of the methodological description, it can be noted that this review concentrated on research studies that investigated ESG’s safety or effectiveness. Also, there was the inclusion of any form of treatment comparison, including comparison with other endoscopic or surgical treatments, medical or diet treatment, and sham endoscopy. In relation to primary outcomes that the included studies were expected to have reported, weight loss formed the focal point. Hence, the studies were expected to have reported aspects such as a loss of excess BMI, percentage loss of excess weight, percentage loss of total weight, and absolute weight loss. Hence, the studies that were included were those reporting at least one of these outcome measures. Apart from primary outcomes, there was a further focus on secondary outcome reporting. The primary interest was in studies reporting adverse events’ severity and frequency following ESG implementation, aimed at informing the safety and efficacy of the procedure. Also, studies that had reported some of the risk factors for patients and the ESG procedure that could yield adverse events were included.

Another crucial factor that influenced the articles’ inclusion or exclusion criteria involved changes in morbidity. Specific parameters linked to morbidity included changes in health-related quality of life, the need for anti-diabetic treatment or otherwise, reduced sleep apnea or otherwise, and reduced hypertension or otherwise.

Sources and Search Strategy

**Figure 1:**
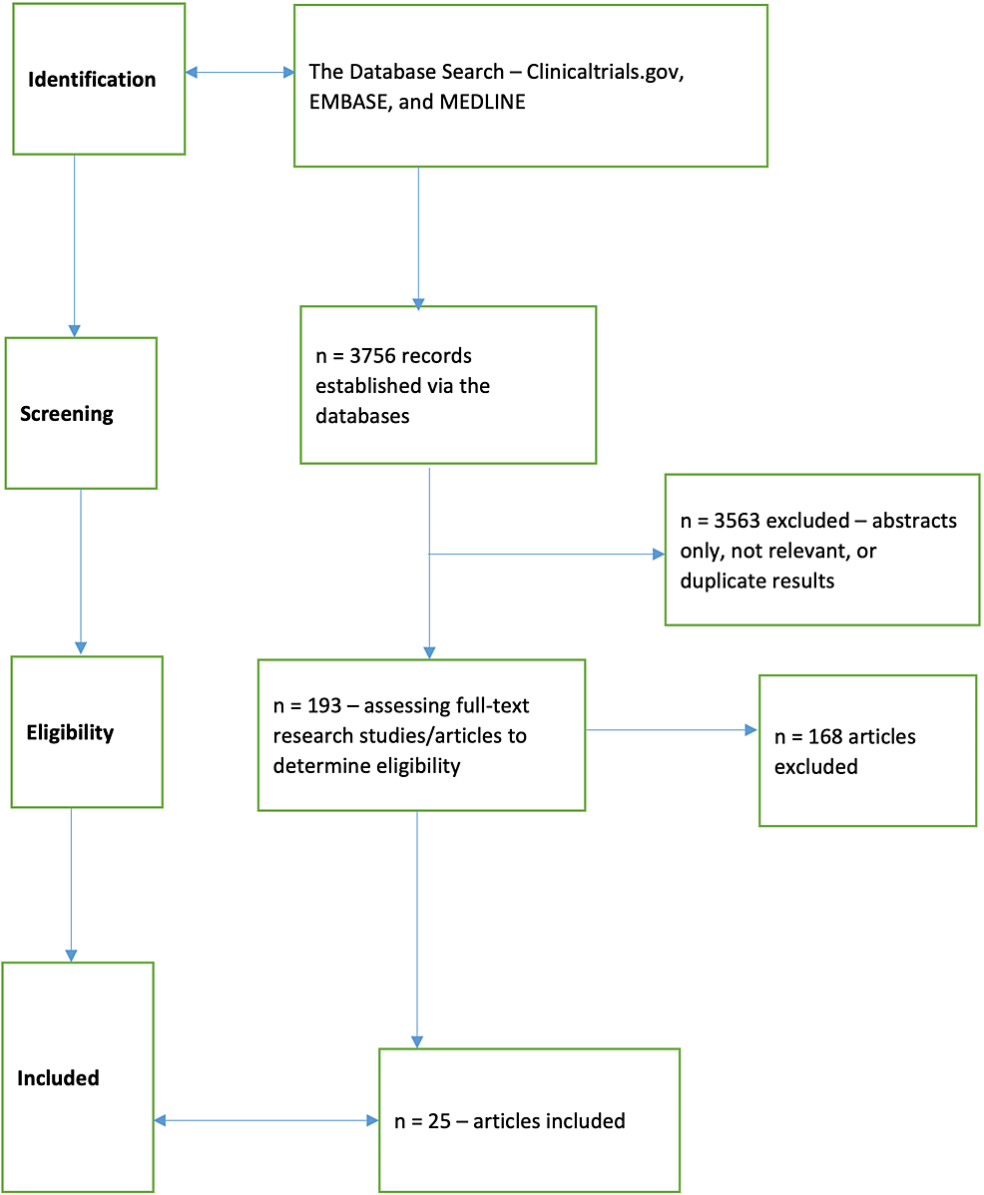
A flowchart for the search strategy and eligible study selection Embase: Excerpta Medica Database; MEDLINE: Medical Literature Analysis and Retrieval System Online

Some of the specific databases that were consulted included Clinicaltrials.gov, Embase (Excerpta Medica Database), and MEDLINE (Medical Literature Analysis and Retrieval System Online, or MEDLARS Online), with the search occurring in January 2023. In the selection process, there was an independent screening of the titles and abstracts that resulted from the search strategy, with the authors then assessing full article reports to discern their satisfaction prior to their final inclusion in the literature review. For any uncertainties that arose regarding whether or not an article satisfied the inclusion criterion, such disagreements on article eligibility were addressed through consensus on the part of the authors. The Preferred Reporting Items for Systematic Reviews and Meta-Analyses (PRISMA) framework was used to uncover the articles established during the initial database search, followed by the selection and review of relevant articles in the screening process, eventually discerning those that proved eligible for inclusion in the current study. Figure 1 summarizes the PRISMA framework-led search strategy and article exclusion and inclusion criteria.

Results

In the literature, one of the areas that have received scholarly attention is the degree of effectiveness of ESG. Using case-matched investigations, there has been a direct comparison of the effectiveness of ESG against high-intensity diet and lifestyle therapy [9]. In one such investigation, the outcomes of 105 individuals who went through the ESG procedure were compared with those of 281 individuals who went through a combination therapy involving high-intensity diet and lifestyle therapies such as behavioral therapy and increase in physical activity. The objective was to discern the efficacy of the two forms of interventions relative to their respective control groups. In the findings, the group exposed to ESG was affirmed to exhibit a total body weight loss mean percentage that was significantly greater compared to the group associated with the combination of lifestyle therapy with a high-intensity diet [9]. With the duration of the investigation stretching to three months and 12 months, the results for the ESG-only group demonstrated a total body weight loss mean percentage of 14% and 20.6%, respectively, compared to 11.3% and 14.3%, respectively, as the total body weight loss mean percentage for the group undergoing a combination of lifestyle therapy with high-intensity diet. From these findings, the efficacy of ESG was clearly demonstrated, thus indicating its superiority in terms of serving as a valuable alternative for patients exhibiting poor compliance to the combination of lifestyle therapy and high-intensity diet. Whether or not the differences in the sample sizes of the ESG-only group versus the group with combination therapy might have affected the final results remains a dilemma.

The efficacy and safety of ESG have also been investigated in patients deemed to be eligible for bariatric surgery but exhibiting BMI less than 40 or comorbidities [10]. Across the stomach’s greater curvature, multi-site sutures were used to achieve gastric tubularization, during ESG with gastric emptying determined through a post-operative water-soluble swallow test. With a period of 18 months used as a mean follow-up duration for the experimental study, values of 39 ± 27 and 11 ± 7 were obtained as the percentage of excess weight and total body weight loss mean percentage, respectively. Also, better results were reported in patients whose extension of the gastric sleeve was over one-third the stomach’s length [10]. As such, an emerging inference is that in high-risk surgical patients, especially those with low BMI and prone to comorbidities, ESG emerges as a safe and effective intervention. However, whether similar results would be obtained if the investigations concentrated on longer follow-up periods and also larger numbers of patients remains a dilemma. In another meta-analysis, 11 studies were used, with a focus on 2,170 patients as the experimental subjects. Focussing on the total body weight loss pool mean percentages following ESG implementation, the values that were documented at 18, 12, and six months were 73%, 60%, and 55.8%, respectively [11]. It is also notable that in a related meta-analysis, eight studies were used and the focus was on 1,859 patients undergoing the ESG procedure. At 24, 12, and six months, the total body weight loss mean percentage values included 60.4%, 61.8%, and 55.8%, respectively [12]. These results suggest the reproducibility of ESG around the world, particularly by demonstrating effective weight loss achievement. However, it should be noted that most of these meta-analyses and systematic reviews fail to clarify some of the adjuvant treatments such as pharmacotherapy or nutritional care in the course of follow-up, implying that whether or not they depict high-level evidence remains unknown. Also, any impact of the sample sizes, disease severity in the participants, and the duration of experimentation is not reported vividly, pointing to the criticality of further clarification of such adjuvant aspects.

ESG’s safety and efficacy have also been investigated from the perspectives of six- and 12-month impacts on weight-related quality of life, safety, and weight loss efficacy. The experimental subjects have been exposed to adjuvant multidisciplinary support before and after the ESG procedure for a period stretching to 12 months [13]. Conducted from the perspective of two-arm prospective cohort research, the key variable that has been used to inform ESG’s efficacy and effectiveness has been the attribute of percentage excess weight loss. Some of the additional variables that have been used to give further insight into ESG’s effectiveness include adverse events, hepatic biochemistry, lipid levels, X-ray absorptiometry, and body composition in terms of features such as the bone mineral content, android-to-gynoid ratio, fat-free mass, and fat mass. In the findings, it has been inferred that ESG is an effective and safe treatment for weight loss in obese adult groups, with the parameter of multidisciplinary support playing a complementary role in yielding this intersection [13]. Hence, patients electing the ESG have been affirmed to be better placed to maintain fat-free mass at six months. However, whether this impact could be longer-term remains unaddressed, pointing to the criticality of more experimental studies focusing on patients with similar demographics but for a longer period of investigation.

In additional studies, the focus has been on ESG’s mechanisms responsible for weight loss. These studies have acknowledged that following the implementation of the ESG procedure, not all patients are likely to respond well. As such, determining some of the factors predicting how patients respond could pave the way for selecting subjects for ESG application or implementation and also inform how peri-procedural care could be implemented. In one of the studies, it has been affirmed that after ESG, during follow-up, high total body weight loss could arise due to factors such as the number of cases, the experience of the endoscopist, compliance with the scheduled visits, high total body weight at one month following ESG, and young age [14]. Similar findings have demonstrated that the total body weight loss is likely to be higher if the experience of an endoscopist exceeds 35 years, especially in individuals or subjects of a young age [15], with the male sex found in additional scholarly investigations to serve as a predictive factor likely to lead to the realization of more than 10% of the total body weight loss at half a year, but not at one year, implying that this factor impacts ESG’s effectiveness but on short-term [16]. Table 1 gives additional insight into some of the scholarly findings that have been documented relative to the mechanism, efficacy or effectiveness, and safety of ESG.

**Table 1:** A review of recent studies on endoscopic sleeve gastrectomy implementation GERD: gastroesophageal reflux disease; ESG: endoscopic sleeve gastrectomy

Reference	Study aim	Results	Clinical Implications
Angrisani et al. (2018) [1]	To report the types and number of bariatric procedures conducted in 2016 in the world and predict surgical trends.	Sleeve gastrectomy was found to be the most performed primary bariatric procedure.	The number of surgical bariatric procedures is increasing, with sleeve gastrectomy dominating.
Brunaldi et al. (2019) [2]	To discuss trends in endoscopic procedure implementation for metabolic syndrome, diabetes, and overweight.	ESG was found to be increasing in implementation trend.	In mildly obese patients, the effectiveness of ESG in steering weight loss was ascertained.
Dayyeh et al. (2013) [3]	To find out the technical feasibility of sleeve gastrectomy in obesity treatment.	The novel technique’s technical feasibility was demonstrated.	There is a need to adopt the ESG procedure in obesity treatment because of its associated feasibility.
Lopez-Nava et al. (2020) [4]	To determine the impact of ESG on metabolic and gut hormones	Following ESG implementation, there is a decrease in the levels of insulin and leptin. Particularly, ESG leads to marked improvements in the patterns of insulin secretion.	The study pointed to the need for further research on the impact of ESG on the peptide-YY, glucagon-like peptide, and fasting ghrelin levels.
Cameron et al. (2012) [5]	To discern the impact of ESG on health-related quality of life.	ESG’s effectiveness in obesity mitigation was ascertained.	Obesity management via ESG tends to attract improvements in health-related quality of life, hence the need to identify patients with poor health-related quality of life to assess eligibility for ESG.
Angrisani et al. (2018) [6]	To determine the correlation among types of endoluminal and bariatric interventions.	ESG was found to be a dominant procedure.	The safety and efficacy of ESG were documented, hence the need for future adoption of the procedure.
Gadiot et al. (2017) [7]	To present follow-up results for comorbidity, failure rate, and weight loss following ESG implementation.	Bariatric surgery proved effective in weight loss management.	High-volume study data is key to making more informed conclusions and inferences.
Lauti et al. (2016) [8]	To find out trends in weight regain after sleeve gastrectomy implementation.	Sleeve dilation and initial sleeve size were found to be the key causes of weight regain.	Adequate follow-up support is key to weight regain minimization.
Cheskin et al. (2020) [9]	To determine changes in weight following ESG implementation.	The mean percentage of the total body weight loss was found to be promising, standing at 14.0%.	ESG is evolving as a valuable alternative for weight loss realization.
Polese et al. (2022) [10]	To evaluate ESG’s efficacy and safety.	In high-risk surgical patients with a BMI of less than 40, ESG was found to be safe and effective.	The need to implement ESG was ascertained.
de Miranda Neto et al. (2020) [11]	To offer an update on ESG’s safety and efficacy.	ESG was affirmed to be an effective and safe procedure relative to therapeutic intervention for primary obesity.	ESG comes with short- and mid-term results.
Singh et al. (2020) [12]	To evaluate ESG procedural technique and safety and efficacy.	ESG comes with minimal cases of mortality and serious adverse events.	ESG remains reproducible among centers globally, coming with weight loss effectiveness and safety profile outcomes that are favorable.
Carr et al. (2022) [13]	The aim was to ascertain patient-centered outcomes and ESG’s safety and efficacy.	ESG as a weight loss treatment was observed to be safe and effective for obese adults.	Multidisciplinary support makes the beneficial effects of ESG more vivid.
Sharaiha et al. (2020) [14]	To assess ESG’s long-term efficacy for obesity treatment.	ESG was found to be effective and safe, with results proving long-term and durable for 5 or more years following the procedure.	ESG is worth considering as a reliable alternative for treating obesity.
Sharaiha et al. (2017) [15]	The study aimed at evaluating the impact of ESG on obesity-related comorbidities and total body weight loss.	ESG proved to be not only minimally invasive but also effective relative to weight loss intervention.	With ESG-reducing markers of obesity-related comorbidities, it is worth considering as a viable option for use with obese patients.
Barrichello et al. (2019) [16]	To understand the technical aspects of ESG and determine its cost-effectiveness.	ESG was found to reduce adipose tissue from baseline significantly, with serious adverse events occurring only in 1.03% of cases.	The effectiveness, safety, and feasibility of ESG were ascertained, hence the need for its future adoption.
Abu Dayyeh et al. (2017) [17]	To determine altered anatomical configuration following ESG induction and the impact on loss of weight	ESG alters metabolic and gut hormones, increases early satiation, and delays gastric emptying	ESG efficacy is affirmed, but the long-term mechanisms of weight loss remain a debate
Jirapinyo et al. (2017) [18]	To ascertain the impact of ESG on the gastric fundus	The gastric fundus stores food, acting as a reservoir. Findings demonstrated that ESG leaves neuronal innervation intact, as well as delays food transit.	ESG is a promising procedure through which early satiety could be stimulated especially through stomach-brain signals.
McCarty et al. (2018) [19]	To find out the emptying time following ESG, hence efficacy.	ESG implementation leads to a notable increase in gastric emptying while delaying solid food emptying.	ESG-based food retention causes early termination of meals while also yielding food intake reductions. Therefore, changes in gut hormones following ESG implementation explain weight loss achievement, with other beneficial effects entailing reduced time to satiation and altered gastric emptying.
Lopez-Nava et al. (2014) [20]	To determine the efficacy and safety of ESG in obesity treatment	Following ESG, no adverse events were reported and hospital stay was less than 24 hours. Furthermore, there was a mean body weight reduction of 8.2±2.5 kg at 1 month, P<0.05; 13.6±4.8 kg at 3 months; P<0.05, and 19.3±9.8 kg at 6 months; P<0.05	The study concludes that ESG could be an effective and safe option for treatment of obesity.
Li et al. (2020) [21]	To determine the safety of ESG	ESG leads to improvements in most symptoms.	Despite the safety of the procedure, some mild, short-term adverse outcomes are evident, including vomiting, nausea, and abdominal pain.
de Moura et al. (2020) [22]	To determine the safety of ESG from the perspective of associated infections.	Following the performance of full-thickness sutures, bacterial translocation and gastric contents could yield intraperitoneal contamination.	The study suggested the need for antibiotic prophylaxis usage at least an hour prior to ESG.
Hedjoudje et al. (2020) [23]	To ascertain ESG safety in patients diagnosed with obesity	Micro perforations and large perforations could result from excessive tension that occurs at the suture site following tissue tearing.	Whereas ESG may cause the formation of an abscess or the collection of perigastric fluids, administering antibiotics could resolve the problem and, in rare cases, surgical procedures or occasional radiologic techniques.
Mohan et al. (2020) [24]	To demonstrate the correlation between ESG and adverse events	Severe adverse events from the perspective of a pooled rate were reported to stand at 2.2%. Specific adverse events included nausea, pain, pulmonary embolism, and epigastric collection or leak.	This study demonstrated the importance of conservative management of adverse events following the ESG procedure.
Asokkumar et al. (2020) [25]	To find out the incidence rate of GERD relative to the implementation of the ESG procedure	There was a significantly lower GERD new-onset incidence following the application of ESG.	Lower rates of GERD after ESG implementation accrue from the ability of the procedure to leave the stomach’s fundus intact, as well as maintain the stomach’s neuronal innervation.

Discussion

Around the world, some of the significant health problems with which populations continue to grapple include metabolic conditions linked to obesity, which include dyslipidemia, type 2 diabetes, and hypertension. To control obesity, bariatric surgery has evolved as one of the most effective techniques, with the effectiveness exceeding that of pharmacologic, exercise, and dietary approaches in most cases. Despite the fact that bariatric surgeries like LSG result in advantages such as higher weight loss, a lower proportion of eligible individuals actually receive the procedure due to issues such as procedure-related complications and the financial burden of the surgery. As such, a promising alternative modality has emerged in the form of ESG. The ESG approach’s uptake has been affirmed to be increasing due to the ease of accessibility as informed by the fact that no incision is needed and also the benefit of low financial burden [15]. Through ESG, the size of the gastric reservoir is reduced in which an OverStitch™ (Apollo Endosurgery, Inc., Austin, Texas, United States) or other full-thickness endoscopic suturing device is used. With the posterior and anterior stomach walls stitched together, the resulting structure becomes tubular.

When total body weight loss is achieved following treatment for obesity, metabolic diseases related to obesity may also be improved. This review has established that in most studies, following ESG implementation, there have been significant mean percentages of total body weight loss. Relative to these threshold values, it is evident that ESG is a beneficial procedure. Indeed, most of the studies have confirmed that ESG as one of the endoscopic bariatric procedures could lead to marked reductions in comorbidities linked to obesity. It is also notable that lower quality of life has been associated with obesity compared to the remainder of the general population. With ESG implementation, however, the weight loss that results tends to be significant, as well as improved health-related quality of life on the part of patients. In patients whose physical inactivity is at baseline and the initial BMI is high, the study has established the efficacy of ESG as per the scholarly findings documented in the literature. Such improvements in the quality of life of patients can be seen to be attributed to ESG’s alteration of the gastrointestinal symptom subdomain.

At this point, it can be seen that ESG has evolved as a primary and safe primary intervention as part of endoscopic bariatric procedures because of its satisfaction of the expected minimal thresholds of both adverse events and total body weight loss. This inference accrues from a position in which more and more evidence supports the safety and efficacy of ESG. For obese persons, therefore, ESG is worth considering as an option, with a particular emphasis on populations diagnosed with mild-to-moderate disease. It is further evident that endoscopists are better placed to play a leading role in spearheading obesity treatment in the future.

## Conclusions

This was a review of the literature on ESG procedural implementation. The specific objective was to uncover the efficacy and safety of the procedure when applied to human subjects. While EGS is generally considered safe, potential risks include bleeding, infection, and perforation of the stomach. The key sources of information were electronic databases, and the criteria for including or excluding studies were clearly defined. These criteria included the years of research covered, the duration of the studies consulted, the characteristics of the study participants, and the topic of investigation discussed in each article. From the findings, it is evident that ESG is an effective procedure when applied to obese populations. In the future, however, there is a need to focus more on randomized controlled trials to discern the long-term efficacy and safety of ESG, as well as uncover the associations between the procedure’s cost-effectiveness, procedure time, and impact on the length of hospital stay.

## References

[1] Angrisani L, Santonicola A, Iovino P (2018). IFSO worldwide survey 2016: primary, endoluminal, and revisional procedures. Obes Surg.

[2] Brunaldi VO, Galvao Neto M (2019). Endoscopic techniques for weight loss and treating metabolic syndrome. Curr Opin Gastroenterol.

[3] Abu Dayyeh BK, Rajan E, Gostout CJ (2013). Endoscopic sleeve gastroplasty: a potential endoscopic alternative to surgical sleeve gastrectomy for treatment of obesity. Gastrointest Endosc.

[4] Lopez-Nava G, Negi A, Bautista-Castaño I, Rubio MA, Asokkumar R (2020). Gut and metabolic hormones changes after endoscopic sleeve gas­troplasty (ESG) vs. laparoscopic sleeve gastrectomy (LSG). Obes Surg.

[5] Cameron AJ, Magliano DJ, Dunstan DW, Zimmet PZ, Hesketh K, Peeters A, Shaw JE (2012). A bi-directional relationship between obesity and health-related quality of life: evidence from the longitudinal AusDiab study. Int J Obes (Lond).

[6] Angrisani L, Santonicola A, Iovino P, Ramos A, Shikora S, Kow L (2021). Bariatric surgery survey 2018: similarities and disparities among the 5 IFSO chapters. Obes Surg.

[7] Gadiot RP, Biter LU, van Mil S, Zengerink HF, Apers J, Mannaerts GH (2017). Long-term results of laparoscopic sleeve gastrectomy for morbid obesity: 5 to 8-year results. Obes Surg.

[8] Lauti M, Kularatna M, Hill AG, MacCormick AD (2016). Weight regain following sleeve gastrectomy-a systematic review. Obes Surg.

[9] Cheskin LJ, Hill C, Adam A (2020). Endoscopic sleeve gastroplasty versus high-intensity diet and lifestyle therapy: a case-matched study. Gastrointest Endosc.

[10] Polese L, Prevedello L, Belluzzi A, Giugliano E, Albanese A, Foletto M (2022). Endoscopic sleeve gastroplasty: results from a single surgical bariatric centre. Updates Surg.

[11] de Miranda Neto AA, de Moura DT, Ribeiro IB (2020). Efficacy and safety of endoscopic sleeve gastroplasty at mid term in the management of overweight and obese patients: a systematic review and meta-analysis. Obes Surg.

[12] Singh S, Hourneaux de Moura DT, Khan A, Bilal M, Ryan MB, Thompson CC (2020). Safety and efficacy of endoscopic sleeve gastroplasty worldwide for treatment of obesity: a systematic review and meta-analysis. Surg Obes Relat Dis.

[13] Carr P, Keighley T, Petocz P (2022). Efficacy and safety of endoscopic sleeve gastroplasty and laparoscopic sleeve gastrectomy with 12+ months of adjuvant multidisciplinary support. BMC Prim Care.

[14] Sharaiha RZ, Hajifathalian K, Kumar R (2021). Five-year outcomes of endoscopic sleeve gastroplasty for the treatment of obesity. Clin Gastroenterol Hepatol.

[15] Sharaiha RZ, Kumta NA, Saumoy M (2017). Endoscopic sleeve gastroplas­ty significantly reduces body mass index and metabolic complications in obese patients. Clin Gastroenterol Hepatol.

[16] Barrichello S, Hourneaux de Moura DT, Hourneaux de Moura EG (2019). Endoscopic sleeve gastroplasty in the management of overweight and obesity: an international multicenter study. Gastrointest Endosc.

[17] Abu Dayyeh BK, Acosta A, Camilleri M, Mundi MS, Rajan E, Topazian MD, Gostout CJ (2017). Endoscopic sleeve gastro­plasty alters gastric physiology and induces loss of body weight in obese individuals. Clin Gastroenterol Hepatol.

[18] Jirapinyo P, Thompson CC (2017). Endoscopic bariatric and metabolic thera­pies: surgical analogues and mechanisms of action. Clin Gastroenterol Hepatol.

[19] McCarty TR, Garg R, O'Brien CR (2018). Efficacy and safety of endoscopic sleeve gastroplasty: a systematic review and metaanalysis. Gastrointest Endosc.

[20] Lopez-Nava G, Galvão MP, da Bautista-Castaño I, Jimenez A, De Grado T, Fernandez-Corbelle JP (2015). Endoscopic sleeve gastroplasty for the treatment of obesity. Endoscopy.

[21] Li P, Ma B, Gong S, Zhang X, Li W (2020). Efficacy and safety of endoscopic sleeve gastroplasty for obesity patients: a meta-analysis. Surg Endosc.

[22] de Moura DT, Badurdeen DS, Ribeiro IB, Leite EF, Thompson CC, Kumbhari V (2020). Perspectives toward minimizing the adverse events of endoscopic sleeve gastroplasty. Gastrointest Endosc.

[23] Hedjoudje A, Abu Dayyeh BK, Cheskin LJ (2020). Efficacy and safety of endoscopic sleeve gastroplasty: a systematic review and meta-analysis. Clin Gastroenterol Hepatol.

[24] Mohan BP, Asokkumar R, Khan SR (2020). Outcomes of endoscopic sleeve gastroplasty; how does it compare to laparoscopic sleeve gastrectomy? A systematic review and meta-analysis. Endosc Int Open.

[25] Asokkumar R, Babu MP, Bautista I, Lopez-Nava G (2020). The use of the Over­Stitch for bariatric weight loss in Europe. Gastrointest Endosc Clin N Am.

